# First insights into the intestinal microbiota of the endangered Angler Saddleback pig

**DOI:** 10.1371/journal.pone.0345650

**Published:** 2026-04-17

**Authors:** Stéphanie Céline Hornburg, Marvin Suhr, Corinna Bang, Dirk Hinrichs, Stefanie Klingel, Georg Thaller, Anna Olschewsky

**Affiliations:** 1 Institute of Animal Nutrition and Physiology, Christian-Albrechts-University Kiel, Kiel, Germany; 2 Institute of Clinical Molecular Biology, Christian-Albrechts-University Kiel, University Hospital Schleswig-Holstein, Kiel, Germany; 3 Animal Breeding Section, Faculty of Organic Agricultural Sciences, University of Kassel, Witzenhausen, Germany; 4 Center for Rare and Endangered Domestic Animals, Warder, Germany; 5 Institute of Animal Breeding and Husbandry, University of Kiel, Kiel, Germany; Sun Yat-Sen University, CHINA

## Abstract

The Angler Saddleback pig is an endangered local breed from northern Germany, characterized by high genetic diversity and exceptional meat quality, but with reduced growth performance compared to commercial pigs. As intestinal microbiota is known to influence health, metabolism, and fat deposition, this study aimed to provide the first characterization of the gut microbial community of this breed. Fecal samples from 37 Angler Saddleback pigs, raised under semi-controlled conditions and fed a restricted diet supplemented with grass-clover silage, were collected at slaughter. Bacterial community composition was analyzed by 16S rRNA gene amplicon sequencing and evaluated using QIIME2, followed by statistical analyses of alpha and beta diversity. The intestinal microbiota was highly diverse, with Bacteroidota (49.5%) and Firmicutes (34.3%) as the dominant phyla, complemented by Spirochaetota and Proteobacteria. Core taxa included *Lactobacillus amylovorus*, *Prevotella*, and Streptococcus, while strong inter-individual variability was observed. Beta diversity analysis revealed significant effects of breeder and fattening period on microbial composition, whereas sex, age, and maternal lineage showed no significant influence. These findings suggest that both diet and early-life environment shape the intestinal microbiota of Angler Saddleback pigs. The study provides a foundation for exploring microbiota-associated traits that may contribute to conservation and breeding strategies for this endangered pig breed.

## Introduction

The extensive use of commercial, fast-growing pigs has led to a decline in the use of local breeds in recent decades [1]. This also applies to the Angler Saddleback (AS) pig, which is a local breed originating from the North of Germany. The AS pig is characterized by higher fat and lower leanmeat contents compared to commercial breeds, which is not consistent with the preferences of consumers, and thus has experienced a drastic decrease in importance for local meat producers [2,3]. Consequently, the breed is now considered endangered with only 115 sows and 7 boars left [4]. The AS pig is described as being robust and healthy, low demanding in terms of feeding and making good use of roughage [3,5]. Additionally, because of their higher intramuscular fat content, their meat is prized by connoisseurs [2]. However, the slower growth and less targeted selection of this breed results in a reduced performance in terms of daily weight gain or feed conversion as compared to commercial pig breeds [3]. Current research identified the intestinal microbiome as important parameter influencing pig health and immunity [6], as well as fiber degradation and lipid metabolism. According to Bergamaschi et al. [7], increasing feed efficiency is correlated with increased α-diversity, as well as with specific bacterial genera, such as *Lactobacillus*, *Blautia*, *Dorea*, and *Eubacterium*. Another study found the taxa *Prevotella, Treponema, Bacteroides*, and *Clostridium* significantly associated with the intramuscular fat amount of pigs [8]. The transfer of lipogenic traits from Jinhua and Landrace pigs to antibiotic-treated mice through fecal microbiota transplantation highlighted the role of the intestinal microbiota in regulating fat metabolism [9], which has also been demonstrated by Qi et al. [10] and Xie et al. [11]. Several studies also investigated the role of dietary fiber and its interaction with the intestinal microbiota in pigs [12–19]. Pig breed and host genome influence the composition of the microbiome with further implications for health and metabolism. Aliakbari et al. [20] found differences in α-diversity of different fast growing pig breeds, with *Lactobacillus* as well as *Prevotella* having the largest differences in abundance. The commercial pig breeds investigated by Bergamaschi et al. [7] differed in the abundance of *Catenibacterium* and *Clostridium* as well as *Bacteroides*. According to Hu et al. [21] local Chinese pig breeds had a lower risk for antimicrobial resistance phenotypes as compared to commercial pigs and this was associated with a lower abundance of *Escherichia coli*. Slower growing pig breeds investigated by Guevarra et al. [22] showed a lower *Firmicutes*/*Bacteroidetes* ratio as compared to commercial pigs. In contrast to that, Yang et al. [23] found that *Firmicutes* were more abundant in the local pig breed, while *Bacteroidetes* were significantly less represented. It is therefore logical to hypothesize that deviations in microbial colonization are at least partially associated with the unique characteristics of the health, growth performance and fat metabolism of the AS pig. Given that many of the positive attributes of the AS pig have not yet been the subject of scientific research, the present study sought to provide a preliminary characterization of the intestinal microbiota of the AS pig. Therefore, the fecal microbiota of 37 AS pigs described in the study of Olschewsky et al. [3] was analyzed via 16S rRNA amplicon sequencing.

## Materials and methods

### Ethic statement

This study was carried out in strict accordance with the recommendations in the German animal protection act [24]. The protocol was approved by the Animal Welfare Officer of the University of Kiel. During slaughter, the pigs were individually stunned with an electrical gripper, and all efforts were made to minimize suffering.

### Fattening experiment

The fecal samples for the analyses of the microbiota were taken from the first of two consecutive trials that are described in more detail by Olschewsky et al. [3]. For this, 21 females and 19 castrates purebred AS pigs from herdbook-registered parents were fattened between October 2018 and May 2019 at the experimental farm Achterwehr of Kiel University. Animals were kept in concordance with the Animal welfare-farm animal husbandry ordinance [25] under semi-controlled conditions (temperature and ventilation). The solid flooring was straw-bedded and the animals remained in single compartments. To meet the demands of this slower growing breed, the pigs were fed restricted with a diet slightly reduced in energy and protein content as compared to commercial practice. Furthermore, grass-clover silage was offered daily with a range of 1.5 to 3.0 kg FM per pig per day. Further details including the nutrient and energy composition of the diets can be found in the publication of Olschewsky et al. [3]. The 40 pigs were raised by three different breeders. As shown in Table 1, differences between the animals of the three breeders are due to age, carcass and meat quality parameters. However, less differences are seen for carcass weight as two slaughter dates took place per trial, for each of which animals of similar weight were selected. The three breeders delivered pigs from two litters of different dams but the same sire, respectively. The piglets supplied by breeders 1 and 3 were housed under conventional conditions, with standard hygiene measures as well as vaccinations and deworming. The husbandry of breeder 2 is certified organic. The stables at this farm are only cleaned with water without the use of disinfectants, the animals are not vaccinated, are not dewormed and no antibiotics have been used for years. However, the animals were revaccinated and dewormed after delivery to the experimental farm.

**Table 1:** Growth, carcass and meat quality of experimental animals.

	Breeder 1	Breeder 2	Breeder 3
Number of pigs (n)	15	15	10
	mean ± sd	mean ± sd	mean ± sd
Age at start (days)	88.9 ± 17.6	110 ± 6.2	123 ± 25.3
Age at slaughter (days)	314.7 ± 18.0	323 ± 15.3	342 ± 25.5
Carcass weight (kg)	108.5 ± 3.9	107.6 ± 5.8	111.2 ± 3.5
Intramuscular fat (%)	2.6 ± 0.7	2.3 ± 0.6	2.6 ± 0.7
Backfatthickness (mm)	38.9 ± 3.7	31.2 ± 3.5	39.2 ± 3.7
Leanmeat percentage (%)	45.8 ± 3.4	51.9 ± 3.4	44.0 ± 2.6

Age at the start and the end of the fattening trial in days, carcass weight as well as meat quality parameters of 40 Angler Saddleback pigs delivered by three different breeders; parameters are expressed as mean ± sd.

### Slaughter of animals and microbiome sampling

The animals were slaughtered in a small countryside slaughterhouse in accordance with the requirements of the European Council Regulation (EC) No 1099/2009 on the protection of animals at the time of killing. Transportation distance was 25 km and the pigs were handled carefully by staff familiar with the pigs. The pigs were individually stunned with an electrical gripper followed by sticking and bleeding. Scalding and dehairing was done by machine with some manual finishing. Directly after slaughter, samples from the intestinal content from the last third of the rectum of each animal were taken and immediately frozen on dry ice. Samples were transported to the laboratory at Kiel University on the same day and preserved at −80 °C until subsequent analysis.

### DNA extraction and 16S rRNA amplicon sequencing

DNA extraction and 16S rRNA amplicon sequencing was performed at the Institute for clinical molecular biology (IKMB) of Kiel University. A total of 250 mg of intestinal content samples was mixed with 1 ml of InhibitEx buffer in 0.70 mm Garnet Bead tubes. Bacterial cell lysis was performed using a SpeedMill PLUS (Analytik Jena GmbH, Jena, DE) at high speed for 45 seconds. The lysate was then incubated for 5 minutes at 550 rpm and 95 °C. Following incubation, lysed cells were pelleted by centrifugation at 20.000 rcf for 1 minute. The total bacterial DNA extracted from incubation supernatant was subsequently isolated using 200 μl of the supernatant as input for the QIAamp® DNA Fast Stool Mini Kit (Qiagen, USA, Cat. no. 51604), following the manufacturer’s instructions on a QIAcube® automation platform (Qiagen, USA). Additionally, extraction blanks were included to monitor potential contamination during DNA preparation. For sequencing, a one-step polymerase chain reaction (PCR) amplification of the V1–V2 region of the 16S rRNA gene was performed using the forward primer 27F ‘AGAGTTTGATCCTGGCTCAG’ and reverse primer 338R ‘TGCTGCCTCCCGTAGGAGT’ [26], employing a dual-barcoding approach. The final reaction volume of 26 μl included 5.0 μl of 5X Phusion HF buffer, 0.5 μl of dNTPs (10 mM), 0.3 μl of Phusion Hot Start II Polymerase (2 U/μl; Thermo Fisher Scientific), 9.2 μl of H2O, 4 μl of each primer (100 μM), and 3 μl of microbial DNA template. Each PCR reaction plate contained a negative control with nuclease-free water to account for contamination, as well as a reaction with a mock community (ZymoBIOMICS Microbial Community Standard, Cat. no. D6305) as a positive control, consisting of eight bacterial isolates with defined abundances to verify adequate performance. The PCR conditions included an initial denaturation at 98 °C for 30 seconds, followed by 30 cycles of 98 °C for 9 seconds, 55 °C for 1 minute (annealing), and 72 °C for 1.5 minutes (extension). A final extension was performed at 72 °C for 10 minutes. The expected fragment size of 550 bp was confirmed via gel electrophoresis on a 2.0% agarose gel. Sequencing of the V1–V2 region of the 16S rRNA gene amplicons was performed. These included amplicons derived from samples, as well as positive and negative controls. The sequencing was conducted at the Institute of Clinical Molecular Biology (IKMB) at Kiel University. An Illumina MiSeq platform (Illumina Inc., San Diego, CA, USA) was used, employing the MiSeq Reagent Kit v3. The procedure followed the manufacturer’s specifications and the protocol described by Trautmann et al. [27]. This resulted in 2 × 300 bp paired-end reads. The raw sequences generated during the study are stored in the European Nucleotide Archive (ENA) at EMBL-EBI under accession number PRJEB98461 (https://www.ebi.ac.uk/ena/browser/view/PRJEB98461).

### Bioinformatics

The 16S rRNA gene sequences were analysed using the Linux-based version of QIIME2 (Quantitative Insights Into Microbial Ecology 2) [28]. Primer sequences and spacers were removed using Cutadapt with primers 27F (5’-AGAGTTTGATCCTGGCTCAG-3’) and 338R (5’-GCTGCCTCCCGTAGGAGT-3’). Quality filtering, chimera removal, and merging of paired-end reads were conducted using DADA2 [29] with truncation parameters of 270 and 244 for forward and reverse reads, respectively, and a quality truncation threshold of 5. Amplicon sequence variants (ASVs) were retained if they occurred at a minimum frequency of 36 and in at least two samples. Subsequently, samples with fewer than 10 observed ASVs were excluded. Sequencing data from 37 animals remained after quality filtering for statistical analysis. For taxonomic classification, only ASVs identified as bacterial at least to the phylum level were retained, while those assigned to mitochondria or chloroplasts were removed from the dataset. Taxonomic assignment was performed using the SILVA reference database version 138.1 [30].

### Statistics

Statistical evaluation of the data was executed using the computation software R (2021). Alpha diversity of the bacterial community from individual pigs was evaluated by calculating observed ASVs, the Shannon and Simpson index. The relative abundances of taxa were evaluated on phylum and order level. Core taxa analysis was performed on ASV level. Beta diversity of bacterial communities was assessed via unweighted UniFrac distances and visualized in a non-metric multi-dimensional scaling plot.

By using individual information from each animal, we investigated factors affecting the bacterial community structure of our dataset. We tested the following factors for statistical influences on alpha and beta diversity: “sex”, “length of fattening period” (209 vs. 230 days), “dam”, “sire” (or “breeder”) and “age at start” (in months). Analysis began with the definition of an appropriate statistical mixed model [31,32]. The residuals were assumed to be approximately normally distributed and heteroscedastic. These assumptions are based on graphical residual analysis. Based on this model, a pseudo R² was calculated [33] and a two-way ANOVA was conducted, followed by multiple contrast tests [34] in order to compare the several levels of the influence factors. Multivariate analysis of beta diversity in pig gut bacteria communities was applied to unweighted UniFrac distances using the adonis function from the *vegan* package with 999 permutations. Pairwise comparisons were performed using permutation MANOVAs on unweighted UniFrac distances with 10,000 permutations. p-values were adjusted manually using the method ‘holm.’

## Results

### Bacterial community composition of individual pigs

**Fig 1:**
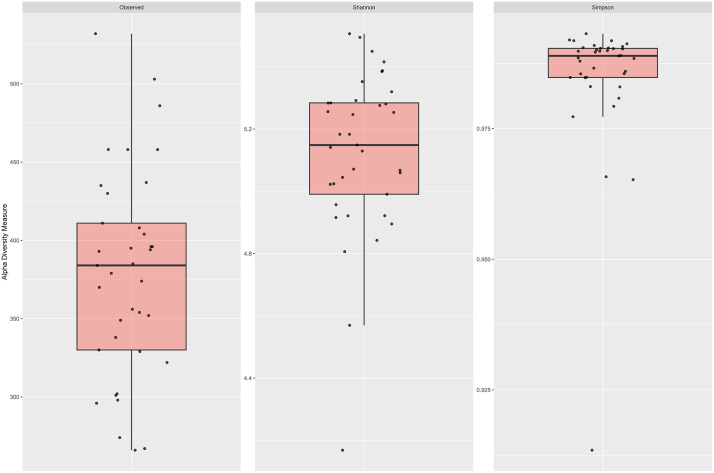
Alpha diversity measures of bacterial community of 37 Angler Saddleback pigs. Observed amplicon sequence variants, Shannon index and Simpson index for each of the individual pigs are shown as singular points. Boxes represent the 25th and 75th percentiles, the black line the median and the whiskers the 1.5 IQR of each dataset.

Alpha-Diversity analysis (Fig 1 and Table 2) demonstrated a high variability between individuals. The number of observed ASVs ranged between 266 and 532, with an average of 378 ASVs and a median number of observed ASVs of 384. Shannon index ranged from 4.2 to 5.5, with an average of 5.1 and a median Shannon index of 5.2. Simpson index showed the lowest inter-individual variability with indices ranging from 0.91 to 0.99.

**Table 2:** Details of alpha diversity measures of bacterial community of 37 Angler Saddleback pigs.

	Observed ASVs	Shannon	Simpson
Min	266.0	4.168	0.9135
Median	384.0	5.149	0.9135
Mean	378.9	5.123	0.9847
Max	532.0	5.506	0.9932

**Fig 2:**
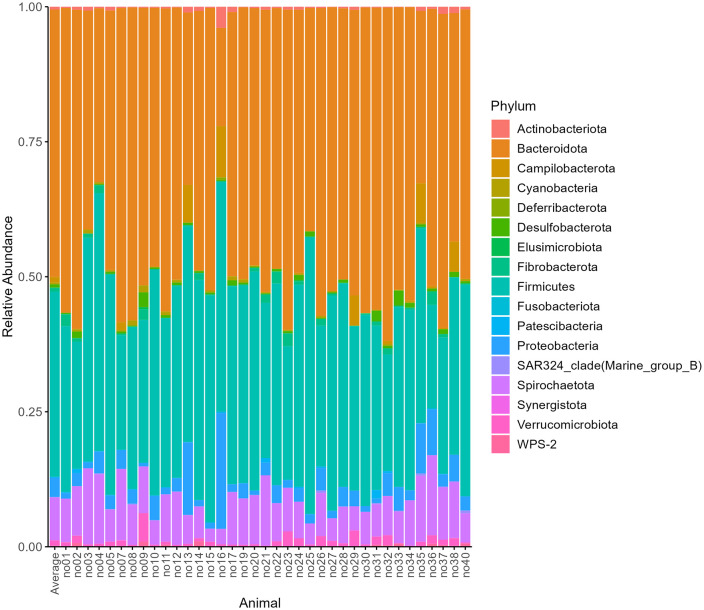
Relative abundance of observed phyla for 37 individual Angler Saddleback pigs. The sample “Average” is a representation of the mean relative abundance among all intestinal content samples.

From the bacterial community analysis among all individual pigs 17 different phyla have been identified (see Fig 2 and S1 Table). On average, Bacteroidota (49.5%) and Firmicutes (34.3%) together accounted for 83.8% of all taxa found in the intestinal content of AS pigs, followed by Spirochaetota (7.95%), Proteobacteria (3.54%), Campilobacterota (1.07%) and Verrucomicrobiota (1.01%). Each of the remaining 11 phyla contributed less than 1% to the bacterial community.

**Fig 3:**
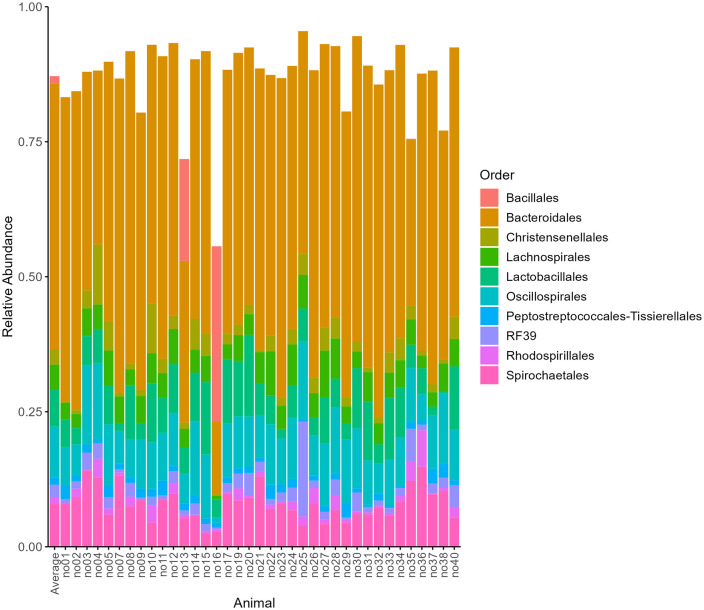
Relative abundance of the top 10 most observed orders for 37 individual Angler Saddleback pigs. The sample “Average” is a representation of the mean relative abundance among all intestinal content samples.

On order level we observed a slightly higher variation between individual pigs and overall, 60 different orders. The most abundant order was Bacteroidales, which accounted for almost half of the bacterial community (49.3%). The rest of the observed orders contributed with less than 10% relative abundance each to the community composition: for example, Oscillospirales (9.4%), Spirochaetales (8.0%), Lactobacillales (6.7%), and Lachnospirales (4.7%) (see Fig 3). Interestingly, a substantial number of orders (47 orders) individually contributed less than 1% to the overall bacterial community composition but 9.1% in total. The order Bacillales contributes with 1.4% to the mean relative abundance, however, this is caused by two pigs (no13 and no16) with a significantly higher relative abundance of Bacillales in comparison to the other animals. All 115 ASVs of the order Spirochaetales belonged either to the genus *Treponema* or *Sphaerochaeta*.

We performed a core taxa analysis to evaluate the distribution of taxa among pig individuals (Table 3). Nine out of ten analysed core taxa could not be assigned on species level. With a prevalence of 1, *Lactobacillus amylovorus*, was found in all pigs with a mean average abundance of 1.9%. Followed by ASVs of the genera *Prevotella* (prv = 0.97), *Prevotellaceae*_NK3B31_group (prv = 0.95), *Streptococcus* (prv = 0.95) and *Muribaculaceae* (prv = 0.92). All core taxa either belong to the orders Lactobacillales or Bacteroidales*.*

**Table 3:** Prevalence and average abundance of 10 core taxa found in 37 Angler Saddleback pig individuals on Phylum, Class, Order, Family, Genus and Species level.

Prevalence	Average Abundance	Phylum	Class	Order	Family	Genus	Species
1.00	1.95%	Firmicutes	Bacilli	Lactobacillales	*Lactobacillaceae*	*Lactobacillus*	*Lactobacillus_amylovorus*
0.97	1.03%	Bacteroidota	Bacteroidia	Bacteroidales	*Prevotellaceae*	*Prevotella*	*uncultured_bacterium*
0.95	1.34%	Bacteroidota	Bacteroidia	Bacteroidales	*Prevotellaceae*	*Prevotellaceae_NK3B31_group*	*uncultured_bacterium*
0.95	3.10%	Firmicutes	Bacilli	Lactobacillales	*Streptococcaceae*	*Streptococcus*	*uncultured_bacterium*
0.92	1.26%	Bacteroidota	Bacteroidia	Bacteroidales	*Muribaculaceae*	*Muribaculaceae*	*uncultured_Porphyromonadaceae*
0.92	1.86%	Bacteroidota	Bacteroidia	Bacteroidales	*p-2534-18B5_gut_group*	*p-2534-18B5_gut_group*	*uncultured_bacterium*
0.92	1.38%	Bacteroidota	Bacteroidia	Bacteroidales	*Prevotellaceae*	*Prevotellaceae_UCG-001*	*uncultured_rumen*
0.89	1.17%	Bacteroidota	Bacteroidia	Bacteroidales	*p-251-o5*	*p-251-o5*	*uncultured_bacterium*
0.86	1.10%	Bacteroidota	Bacteroidia	Bacteroidales	*p-251-o5*	*p-251-o5*	*uncultured_bacterium*
0.81	1.06%	Bacteroidota	Bacteroidia	Bacteroidales	*p-2534-18B5_gut_group*	*p-2534-18B5_gut_group*	*uncultured_bacterium*

**Fig 4:**
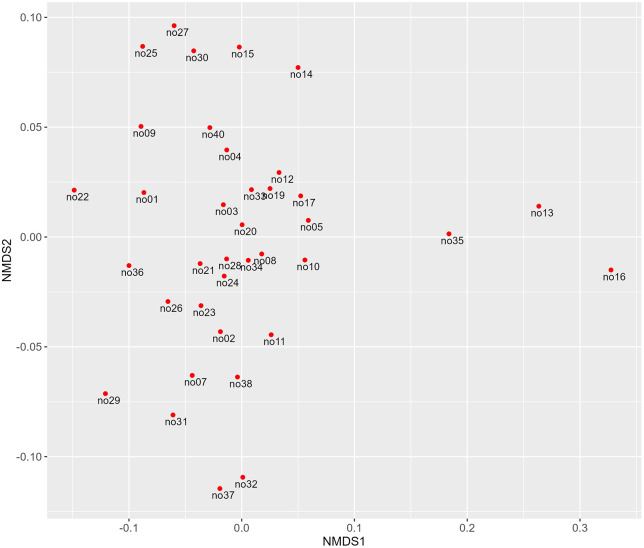
Non-metric multi-dimensional scaling (NMDS) plot of 37 individual Angler Saddleback pigs based on unweighted UniFrac distances. Bacterial community of each pig is displayed via red dots and labelled with pig-ID.

Beta-diversity analysis (Fig 4) revealed an even distribution of the bacterial communities. Three animals, however, (no13, no16 and no35) separated from the rest of the dataset. These individuals already accounted for significantly different abundances of Bacillales, e.g., (see Fig 3).

### Pig characteristics influencing the bacterial community formation

**Fig 5:**
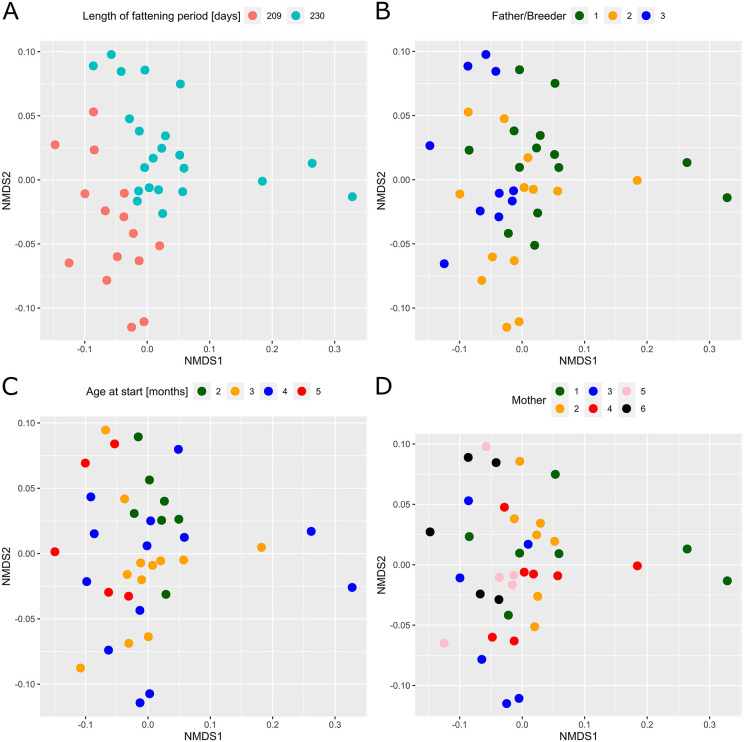
Non-metric multi-dimensional scaling (NMDS) plots of unweighted UniFrac distances of 37 individual Angler Saddleback pigs. Presented are the influencing factors (A) length of fattening period (in days), (B) sire/breeder, (C) age at start of experiment (in months), and (D) dam.

Individual information (sex, length of fattening period, sire or breeder, dam and age at start of experiment) from each animal was used, to identify factors affecting the bacterial community structure of our dataset (Fig 5). It is important to emphasize that the “sire/breeder” factor includes not only host genetics, but also different husbandry, feeding, and health management conditions on the farms of origin. None of the analysed characteristics had an effect on alpha diversity, as Observed ASVs, Shannon-Index and Simpson’s were not significantly different between any of the tested factors. On beta diversity level, however, we observed significant effects of “length of fattening period” (p = 0.001) and of “sire/breeder” (p = 0.002) on the bacterial community structure, without interaction effects (p = 0.486). The overall ANOVA of the factor “dam” was significant (p = 0.003), the effect was, however, gone when pairwise comparisons with p-value adjustment were performed.

## Discussion

### Most abundant orders and potential associations with characteristics of the AS pig

The bacterial gut community of the AS pig appears to be rich and divers, however, 95% of bacterial taxa found in the intestinal content of the pigs belong to the phyla Bacteroidota, Firmicutes, Spirochaetota and Proteobacteria, distributed among the orders Bacteroidales, Oscillospirales, Spirochaetales or Lactobacillales, among others.

**Bacteroidales**, a prominent order within the Bacteroidota phylum, play vital roles in the pig intestine. Species from the order Bacteroidales are known to ferment complex polysaccharides, such as dietary fibres, resulting in the production of short chain fatty acids (SCFAs) like acetate, propionate, and butyrate. These SCFAs serve as energy sources for colonocytes and have anti-inflammatory properties [35]. Members of the Bacteroidales order can further inhibit the colonization of pathogenic bacteria by competing for nutrients and adhesion sites, thus contributing to the maintenance of a balanced gut microbiota [36]. Some Bacteroidales are involved in the synthesis of essential vitamins, such as vitamin K and certain B vitamins, which are vital for the host’s metabolic processes [37]. Bacteroidota are efficient degraders of complex carbohydrates and fibres. In intestinal content samples of AS pigs (this study) eight out of ten core taxa belong to the order Bacteroidales, with *Prevotellacaea* and *Muribaculaceae* being the most prevalent families among the Bacteroidota. *Prevotella,* but also *Treponema* (a Spirochaete)*,* are specifically enriched in herbivorous mammals with increased fibre intake from plant polysaccharides, such as in traditional human populations [38]. In pigs, the abundance of n-butyric acid in the rectal content was positively associated with *Prevotella* spp., suggesting *Prevotella* as a biomarker for SCFA production [39].

**Oscillospirales** is an order within the phylum Firmicutes, encompassing anaerobic, Gram-positive bacteria that are integral components of the gut microbiota in pigs. Members of Oscillospirales, particularly within the family *Oscillospiraceae*, are adept at fermenting complex carbohydrates, leading to the production of SCFAs such as butyrate. Butyrate contributes to gut health and serves as a primary energy source for colon mucosa cells [40]. The abundance of Oscillospirales has been linked to host metabolic traits and growth performance in pigs. Studies indicate that certain taxa within this order may influence nutrient absorption and energy metabolism, thereby affecting growth rates [41]. Interestingly, due to their beneficial roles in gut health and metabolism, Oscillospirales are considered potential candidates for next-generation probiotics. Their ability to produce SCFAs and modulate the immune system positions them as promising agents for enhancing host health [42].

**Spirochaetales** are spiral-shaped, anaerobic bacteria that contribute to both gut function and disease. They colonize the large intestine in a fairly stable manner, potentially participating in low-level fermentation and interacting with the mucosal immune system. Nevertheless, several *Brachyspira* species, for example, are pathogenic, causing colitis/typhlitis or diarrhea [43]. In our study, however, all ASVs of the order Spirochaetales belonged either to the genus *Treponema* or *Sphaerochaeta*. *Sphaerochaeta* have been found to be negatively correlated with body weight, average daily weight gain and backfat thickness in Enshi black pigs, a Chinese indigenous pig breed [44]. In a comparison study of the fecal microbiota from Duroc, Landrace and Yorkshire pigs, *Sphaerochaeta* were one of four significantly differentially abundant genera between the three breeds with highest abundances in Landrace pigs [45]. Interestingly, it has been reported from human populations that Spirochaetes are increasingly absent from populations consuming a westernized diet (low in fibre) [46], supporting our findings of enriched Spirochaetales in AS pigs fed with fibre-rich diets.

**Lactobacillales**, an order of lactic acid bacteria, are prominent members of the pig gut microbiota. These bacteria play multifaceted roles in maintaining intestinal health and overall physiology. Lactobacillales influence the host’s immune responses by interacting with the mucosal immune system, promoting the development of regulatory T cells, and enhancing gut barrier function, which is why Lactobacillales are often supplemented as probiotics [47,48]. Lactobacillales also ferment non-digestible carbohydrates, resulting in the production of SCFAs such as acetate, propionate, and butyrate. By producing lactic acid, Lactobacillales lower the pH of the gut environment, creating conditions that favour the activity of fibre-degrading enzymes and inhibit pathogenic bacteria. This modulation supports efficient fibre fermentation and maintains gut health [47].

*Lactobacillus amylovorus*, a member of the Lactobacillales was prevalent in all of the pig fecal samples (see Table 3). It is well known, that *L. amylovorus* is an abundant species in the small intestine of pigs [49] where it contributes to carbohydrate metabolic functions [50] and intestinal immunity [51]. Moreover, *L. amylovorus* is a potential probiotic, as it exhibits antimicrobial activity against enteric pathogens [52,53] and was found to improve the growth performance of IUGR (Intrauterine growth restriction) piglets by promoting lactose utilization in the small intestine and enhancing intestinal barrier functions [54].

It should be noted that the present study is based on 16S rRNA gene amplicon sequencing, which allows robust characterization of bacterial community composition at higher taxonomic levels but does not provide strain-level resolution or direct functional information. While some taxa, such as *L. amylovorus*, could be assigned at species level, functional traits and metabolic capacities were inferred based on previously published literature rather than directly measured. Consequently, strain-specific effects and functional heterogeneity within taxa cannot be excluded and may play an important role in shaping host–microbiota interactions. Future studies using shotgun metagenomics, metatranscriptomics, or metabolomics would be required to resolve strain-level variation and to directly link microbial functions to host phenotypes in the AS pig.

### The bacterial community composition in comparison to other local and commercial pig breeds

It is noteworthy, that the bacterial community of the AS pigs from the current study is dominated by Bacteroidota (49.5%), instead of Firmicutes (34.3%), which is contrasting the community composition of other local breeds: For example, metagenome shotgun sequencing of three fecal samples from Italian Nero Siciliano pigs revealed that Firmicutes alone was the predominant phylum in all samples with 86% relative abundance on average, followed by Actinobacteria (3.0%), Proteobacteria (∼3.0%), and Bacteroidetes (1.3%) [55]. The three donor pigs used were fattened at three different farms and were fed with a pelleted complete feed, supplemented with different natural plant resources, such as acorn, citrus pulp or pasture. In colon samples of a local Chinese pig breed, the Jiaxing Black pig, also Firmicutes were found to be the predominant phylum, followed by Bacteroidetes and Proteobacteria [56]. Further, in fecal samples of two Iberian purebred pig strains (Retinto and Entrepelado) Firmicutes were the most abundant phylum, with an average relative abundance of 93.9%, followed by Bacteroidota (4.5%) [57]. Those pigs were fed commercial feedstuffs *ad libitum* by automatic feeders. 16S rRNA gene amplicon sequencing of different gut sections from Landrace and Jinhua pigs revealed a dominance of Firmicutes for both, Landrace (90.26%) and Jinhua (86.98%), in colon samples [58]. Interestingly, in cecum samples the relative abundance of Bacteroidetes increased drastically (Landrace: 24.36% and Jinhua: 20.80%).

However, a study investigating the microbiota of a Duroc x Iberian crossbred population found Bacteroidota (44.08%) being the most dominant phylum in rectal content samples, followed by Firmicutes (38.17%) [39]. Those animals were fed *ad libitum* with a standard commercial diet based on barley and wheat. A comparison study of the bacterial community in fecal samples from Duroc, Landrace and Yorkshire pigs, raised under the same nutritional and management conditions of a commercial pig farm, revealed significantly different relative abundances of Firmicutes, Bacteroidota and Spirochaetota between the three different breeds, with highest abundances of Firmicutes in Duroc pigs and highest abundances of Bacteroidota in Yorkshire pigs [59]. The highly increased amount of Bacteroidota in comparison to other local pig breeds could, therefore, either be a specific characteristic of AS pigs or the result of the grass-clover silage, that was offered during the fattening experiment.

However, comparisons of the microbial colonization of AS pigs with other breeds examined in previous studies are limited. As described, the husbandry and feeding conditions, as well as the methods (e.g., bioinformatic pipelines and sequencing regions), varied distinctly, meaning that any differences identified may not be solely due to genetics. Additionally, samples from AS pigs were only taken at slaughter. Since age significantly influences the diversity of the pig microbiome [60], the present study only provides a snapshot at the end of the fattening period. Furthermore, it should be emphasized that the AS pigs were comparatively old with around ten month due to their slower growth. Therefore, the age difference could also be responsible for the observed differences to some extent.

### The influence of piglet nursing environment on the bacterial community structure

Overall, no influence of the tested factors (sex, length of fattening period, sire or breeder, dam and age at start of experiment) on alpha diversity and partly on beta diversity was found. The lack of significance may be related to the limited sample size. While 37 pigs is an adequate size, it is further reduced when analyzing influencing factors, increasing the probability of type II errors.

However, the bacterial community was strongly separated by pig’s birthplace (i.e., breeder with distinct sires), but not by their individual dams, which implies that either the genetics of the sire was driving the bacterial community, or the original environment (including diet) of the piglets had long-lasting effects on the intestinal microbiota. Although piglets from the three breeders had a different starting age when they entered the experiment, the factor age did not significantly influence the bacterial community composition. In a recent study by Tancredi et al. [61] it was shown that the maternal influence on the microbiome development of piglets decreased with age and post-weaning, which could explain the negligible effect of the sows on the pig microbiota at the end of the experiment. In contrast, Tancredi et al. [61] additionally observed that the influence of cage mates increased and the bacterial composition remained highly consistent over time, which would be in line with our observation of a significant breeder effect. Further, diet generally shapes the pig intestinal microbiota as has been observed in several studies, specifically during weaning [62–64]. It has also been shown that early dietary interventions have long-lasting effects on the intestinal microbial community, as well as maternal nutrition [65]. This could explain the separation of the bacterial composition by the factor breeder at the end of the fattening experiment. The piglets underwent different nursing, feeding and weaning strategies on their respective farms before entering the experiment, which was unavoidable due to the small population of AS pigs. However, not only diet shapes the intestinal microbiome of pigs, but also housing and environment, management, social interactions or medical interventions [66], which are all included in the factor “breeder” and significantly differ between farms. These elements could not be separated from each other in the present analysis; therefore, no direct conclusions can be drawn about the influence of AS pig host genetics on the microbiota characteristics. However, an analysis of a large dataset with SNP array genotypes from a representative AS population showed that there are indeed genetic differences among animals from different breeders [67]. This study also included the genomic data of the animals whose microbiomes were examined herein. The results provide further evidence that the environment (e.g., husbandry and feeding) may not be the only relevant component of the “breeder” factor.

## Conclusions

This study provides the first insights into the intestinal microbiota of the endangered AS pig. The bacterial community was found to be diverse, with Bacteroidota and Firmicutes dominating, and core taxa such as *Lactobacillus amylovorus* and *Prevotella* being consistently present. Comparisons with other pig breeds suggest that the high abundance of Bacteroidota may be a unique feature of the AS pig and may be linked to its fiber-rich diet. Moreover, breeder effects indicated that early-life environment and management have a lasting influence on microbial composition, while individual attributes such as sex or age were less relevant. These results highlight the potential of the gut microbiota to contribute to the robustness and fat metabolism typical of this local breed. A deeper understanding of these microbial features may help to identify valuable characteristics (e.g., use of local feed resources) for future breeding programs. Making local breeds profitable based on targeted breeding management is important for long-term conservation. Therefore, unique traits, such as those related to microbial colonization, can play a key role in the conservation of the AS pig breed.

## Supporting information

S1 Table Average relative abundance of observed phyla and orders across all individuals. (DOCX)
