# Supplementary material for: First insights into the intestinal microbiota of the endangered Angler Saddleback pig
Source: PLoS One. 2026 Apr 17;21(4):e0345650. doi: 10.1371/journal.pone.0345650 (PMC13089714; doi:10.1371/journal.pone.0345650)
Supplement: S1 Table — (DOCX) [file pone.0345650.s001.docx]

**First insights into the intestinal microbiota of the endangered Angler Saddleback pig**

Stéphanie Céline Hornburg^1^, Marvin Suhr^1^, Corinna Bang^2^, Dirk Hinrichs^3^, Stefanie Klingel^4^, Georg Thaller^5^, Anna Olschewsky^3^*

^1^ Institute of Animal Nutrition and Physiology, Christian-Albrechts-University Kiel, Kiel, Germany

^2^ Institute of Clinical Molecular Biology, Christian-Albrechts-University Kiel, University Hospital Schleswig-Holstein, Kiel, Germany

^3^ Animal Breeding Section, Faculty of Organic Agricultural Sciences, University of Kassel, Witzenhausen, Germany

^4^ Center for Rare and Endangered Domestic Animals, Warder, Germany

^5^ Institute of Animal Breeding and Husbandry, University of Kiel, Kiel, Germany

*Corresponding author:

E-Mail: olschewsky@uni-kassel.de (AO)

**S1 Table. Average relative abundance of observed phyla and orders across all individuals.**

|  | **Phylum** | **Abundance** |
| --- | --- | --- |
| **1** | Bacteroidota | 0.495 |
| **2** | Firmicutes | 0.343 |
| **3** | Spirochaetota | 0.080 |
| **4** | Proteobacteria | 0.035 |
| **5** | Campilobacterota | 0.012 |
| **6** | Verrucomicrobiota | 0.010 |
| **7** | Fibrobacterota | 0.007 |
| **8** | Desulfobacterota | 0.006 |
| **9** | Actinobacteriota | 0.005 |
| **10** | Cyanobacteria | 0.003 |
| **11** | WPS-2 | 0.002 |
| **12** | Patescibacteria | 0.002 |
| **13** | SAR324_clade(Marine_group_B) | 0.001 |
| **14** | Elusimicrobiota | 0.000 |
| **15** | Deferribacterota | 0.000 |
| **16** | Synergistota | 0.000 |
| **17** | Fusobacteriota | 0 |
|  | **Order** |  |
| **1** | Bacteroidales | 0.493 |
| **2** | Oscillospirales | 0.094 |
| **3** | Spirochaetales | 0.080 |
| **4** | Lactobacillales | 0.067 |
| **5** | Lachnospirales | 0.047 |
| **6** | Christensenellales | 0.027 |
| **7** | RF39 | 0.023 |
| **8** | Bacillales | 0.014 |
| **9** | Peptostreptococcales-Tissierellales | 0.013 |
| **10** | Rhodospirillales | 0.013 |
| **11** | Erysipelotrichales | 0.013 |
| **12** | Acidaminococcales | 0.012 |
| **13** | Campylobacterales | 0.011 |
| **14** | WCHB1-41 | 0.009 |
| **15** | Clostridia_UCG-014 | 0.007 |
| **16** | Veillonellales-Selenomonadales | 0.007 |
| **17** | Fibrobacterales | 0.007 |
| **18** | Burkholderiales | 0.006 |
| **19** | Paracaedibacterales | 0.005 |
| **20** | Bradymonadales | 0.004 |
| **21** | Monoglobales | 0.004 |
| **22** | Clostridiales | 0.003 |
| **23** | Pseudomonadales | 0.003 |
| **24** | Gastranaerophilales | 0.003 |
| **25** | Corynebacteriales | 0.003 |
| **26** | Enterobacterales | 0.002 |
| **27** | Aeromonadales | 0.002 |
| **28** | Clostridia_vadinBB60_group | 0.002 |
| **29** | Flavobacteriales | 0.002 |
| **30** | Coriobacteriales | 0.002 |
| **31** | Desulfovibrionales | 0.002 |
| **32** | WPS-2 | 0.002 |
| **33** | Saccharimonadales | 0.002 |
| **34** | Clostridia | 0.001 |
| **35** | Peptococcales | 0.001 |
| **36** | Mycoplasmatales | 0.001 |
| **37** | Acholeplasmatales | 0.001 |
| **38** | Caulobacterales | 0.001 |
| **39** | SAR324_clade(Marine_group_B) | 0.001 |
| **40** | Xanthomonadales | 0.001 |
| **41** | Izemoplasmatales | 0.001 |
| **42** | Rickettsiales | 0.001 |
| **43** | Verrucomicrobiales | 0.001 |
| **44** | Staphylococcales | 0.001 |
| **45** | Elusimicrobiales | 0.000 |
| **46** | Caldicoprobacterales | 0.000 |
| **47** | Alteromonadales | 0.000 |
| **48** | Micrococcales | 0.000 |
| **49** | uncultured | 0.000 |
| **50** | Actinomycetales | 0.000 |
| **51** | Oligosphaerales | 0.000 |
| **52** | Deferribacterales | 0.000 |
| **53** | Absconditabacteriales_(SR1) | 0.000 |
| **54** | Oceanospirillales | 0.000 |
| **55** | Synergistales | 0.000 |
| **56** | Rhodobacterales | 0.000 |
| **57** | Propionibacteriales | 0.000 |
| **58** | Cardiobacteriales | 0.000 |
| **59** | Fusobacteriales | 0 |
| **60** | Pasteurellales | 0 |
